# Pancreas CT assessment for pancreatic ductal adenocarcinoma resectability: effect of tube voltage and slice thickness on image quality and diagnostic performance

**DOI:** 10.1186/s40644-023-00637-9

**Published:** 2023-12-18

**Authors:** Dong Ho Lee, Seung Soo Lee, Jeong Min Lee, Jin-Young Choi, Chang Hee Lee, Hong Il Ha, Bo-Kyeong Kang, Mi Hye Yu, Won Chang, Sae Jin Park

**Affiliations:** 1 Department of Radiology, Seoul National University Hospital, Seoul, South Korea; 2 Department of Radiology and Research Institute of Radiology, Asan Medical Center, University of Ulsan College of Medicine, Seoul, South Korea; 3 Department of Radiology, Seoul National University College of Medicine, National University College of Medicine, 101 Daehak-ro, Jongno-gu, Seoul, 03080 South Korea; 4 Department of Radiology and Research Institute of Radiological Science, Severance Hospital, Yonsei University College of Medicine, Seoul, South Korea; 5 Department of Radiology, Korea University Guro Hospital, South Korea University Medicine, Seoul, South Korea; 6 Department of Radiology, Hallym University Sacred Heart Hospital, Anyang, South Korea; 7 Department of Radiology, Hanyang University College of Medicine, Seoul, South Korea; 8 Department of Radiology, Konkuk University College of Medicine, Seoul, South Korea; 9 Department of Radiology, Seoul National University Bundang Hospital, Seoul, South Korea

**Keywords:** High-resolution pancreas CT, Pancreatic ductal adenocarcinoma, Resectability assessment

## Abstract

**Objectives:**

To assess the resectability of pancreatic ductal adenocarcinoma (PDAC), the evaluation of tumor vascular contact holds paramount significance. This study aimed to compare the image quality and diagnostic performance of high-resolution (HR) pancreas computed tomography (CT) using an 80 kVp tube voltage and a thin slice (1 mm) for assessing PDAC resectability, in comparison with the standard protocol CT using 120 kVp.

**Methods:**

This research constitutes a secondary analysis originating from a multicenter prospective study. All participants underwent both the standard protocol pancreas CT using 120 kVp with 3 mm slice thickness (ST) and HR-CT utilizing an 80 kVp tube voltage and 1 mm ST. The contrast-to-noise ratio (CNR) between parenchyma and tumor, along with the degree of enhancement of the abdominal aorta and main portal vein (MPV), were measured and subsequently compared. Additionally, the likelihood of margin-negative resection (R0) was evaluated using a five-point scale. The diagnostic performance of both CT protocols in predicting R0 resection was assessed through the area under the receiver operating characteristic curve (AUC).

**Results:**

A total of 69 patients (37 males and 32 females; median age, 66.5 years) were included in the study. The median CNR of PDAC was 10.4 in HR-CT, which was significantly higher than the 7.1 in the standard CT (*P*=0.006). Furthermore, HR-CT demonstrated notably higher median attenuation values for both the abdominal aorta (579.5 HU vs. 327.2 HU; *P*=0.002) and the MPV (263.0 HU vs. 175.6 HU; *P*=0.004) in comparison with standard CT. Following surgery, R0 resection was achieved in 51 patients. The pooled AUC for HR-CT in predicting R0 resection was 0.727, slightly exceeding the 0.699 of standard CT, albeit lacking a significant statistical distinction (*P*=0.128).

**Conclusion:**

While HR pancreas CT using 80 kVp offered a notably greater degree of contrast enhancement in vessels and a higher CNR for PDAC compared to standard CT, its diagnostic performance in predicting R0 resection remained statistically comparable.

## Introduction

Pancreatic ductal adenocarcinoma (PDAC) stands out as one of the most aggressive and lethal malignancies. The estimated 5-year overall survival rate after diagnosis is typically less than 10% [1, 2]. Among the various treatment modalities available for PDAC, complete tumor removal with a negative resection margin (R0) remains the sole potentially curative method, offering a chance for long-term survival [3]. However, the presence of tumor invasion into major vessels such as the celiac axis and superior mesenteric artery, or the occurrence of distant metastasis, renders PDAC patients ineligible for surgical resection. As a result, fewer than 30% of PDAC patients can undergo curative resection at the time of diagnosis [4]. Given this, assessing the eligibility of PDAC for surgical resection becomes a crucial step in the management of PDAC patients.

Among the various imaging modalities for PDAC, computed tomography (CT) has been the most widely used modality, primarily attributed to the high spatial and temporal resolution. This capability enables the meticulous evaluation of PDAC's tumor vascular involvement, a crucial aspect in determining its resectability [5]. To ensure standardized imaging examinations, the current guidelines of the National Comprehensive Cancer Network (NCCN) define a specific CT protocol for assessing PDAC resectability. This protocol typically involves a tube voltage of 120 kVp and a slice thickness (ST) of 3 mm, without any interslice gap, as the standard approach. Retrospective studies conducted previously have demonstrated that this standard pancreas CT protocol achieves an accuracy range of 65-80% in determining PDAC resectability [6, 7]. Nevertheless, despite the adequate application of the standard pancreas CT to PDAC patients, a subset of around 10% of PDAC cases may exhibit iso-attenuating features within the pancreas parenchyma. This arises due to the insufficient attenuation difference between the PDAC and the neighboring pancreatic parenchyma, making the precise assessment of tumor vascular involvement challenging [8, 9]. Consequently, there have been continuous efforts to enhance both the imaging quality and diagnostic efficacy of pancreas CT in the assessment of PDAC resectability.

Prior research has indicated that utilizing a low tube voltage CT (80 kVp) can enhance the contrast enhancement of target tissues by increasing x-ray absorption of iodine. This leads to heightened tumor conspicuity and an improved contrast-to-noise ratio (CNR) compared to the standard 120 kVp CT [10]. Furthermore, employing thin-section CT with a 1 mm reconstruction interval has been shown to offer superior spatial resolution compared to the 3 mm slice thickness. This finer resolution enables a more precise assessment of perineural invasion in PDAC [11]. In this context, the combination of an 80 kVp tube voltage and thin-section reconstruction holds the potential to improve the image quality of pancreas CT scans. However, it remains uncertain whether employing high-resolution (HR) pancreas CT with an 80 kVp setting and thin-section reconstruction can concurrently enhance both image quality and diagnostic accuracy in evaluating PDAC resectability when compared to the conventional standard pancreas CT protocol. This study aims to evaluate the image quality and diagnostic performance of HR-CT in assessing PDAC resectability, relative to the standard protocol for pancreas CT.

## Materials and methods

### study design and patient enrollment

This research constitutes a secondary analysis derived from a multicenter prospective study (Study identifier: NCT03895177). It encompassed patients who underwent both conventional CT and high-resolution CT scans. The study received approval from the institutional review board, and written informed consents were obtained from all participating patients. In the previous multicenter prospective study, a total of 138 PDAC patients were enrolled. These patients underwent HR-CT scans to determine PDAC resectability, which was based on either histopathologic findings following surgery or clinical decisions made after multidisciplinary discussions at six university-affiliated hospitals. From this cohort, the current study focused on patients who had available pancreas CT scans performed using the standard protocol, utilizing a tube voltage of 120 kVp and a reconstruction interval of 3 mm. These scans were conducted within one month from the date of the PDAC resectability determination, and this subgroup was included and analyzed in the current study.

### Imaging acquisition

All participants underwent standard pancreas CT utilizing a tube voltage of 120 kVp and a reconstruction interval of 3 mm, as well as HR-CT using an 80 kVp tube voltage and a 1 mm reconstruction interval. These scans were carried out within one month from the date of PDAC resectability determination. Both pancreas CT protocols encompassed unenhanced scans spanning from the lower thorax, including the liver dome, down to the level of the lower pole of the right kidney. Following the acquisition of the unenhanced scan, an iodinated contrast with a dosage of 600 mg iodine/kg was intravenously administered through an 18G catheter positioned in the antecubital vein using a power injector. The flow rate was maintained at equal to or greater than 2 mL/second. Both pancreas CT protocols encompassed imaging during both the arterial phase and the portal venous phase. To establish the optimal timing for the arterial phase scan, the bolus tracking method was employed. The arterial phase images were obtained 17 seconds after the contrast reached ≥100 Hounsfield units in the proximal abdominal aorta. Subsequently, the portal venous images were acquired 70 seconds after the initiation of contrast injection.

In the standard pancreas CT protocol, the tube voltage was set at 120 kVp with an effective tube current of 150 mAs. The ST for both arterial and portal venous phase images was 3 mm without an inter-slice gap. Coronal reconstruction images for both phases were created using the same ST as the axial images. In the HR-CT protocol, 80 kVp tube voltage was used, and the effective tube current was set at 325 mAs to mitigate image noise stemming from the use of 80 kVp tube voltage. The ST was set at 1 mm without an inter-slice gap for high-resolution CT. Correspondingly, the coronal reformatted images for both phases were also created with the same 1 mm reconstruction interval. Both the standard and HR pancreas CT protocols employed an iterative reconstruction algorithm for the imaging reconstruction process (ADMIRE or SAFIRE level 2 for Siemens scanner; ASiR level 2 for GE scanner; and iDOSE or IMR level 2 for Philips scanner). The median radiation dose of each CT protocol was recorded and compared.

### Imaging analysis

#### Quantitative analysis

**Fig. 1:**
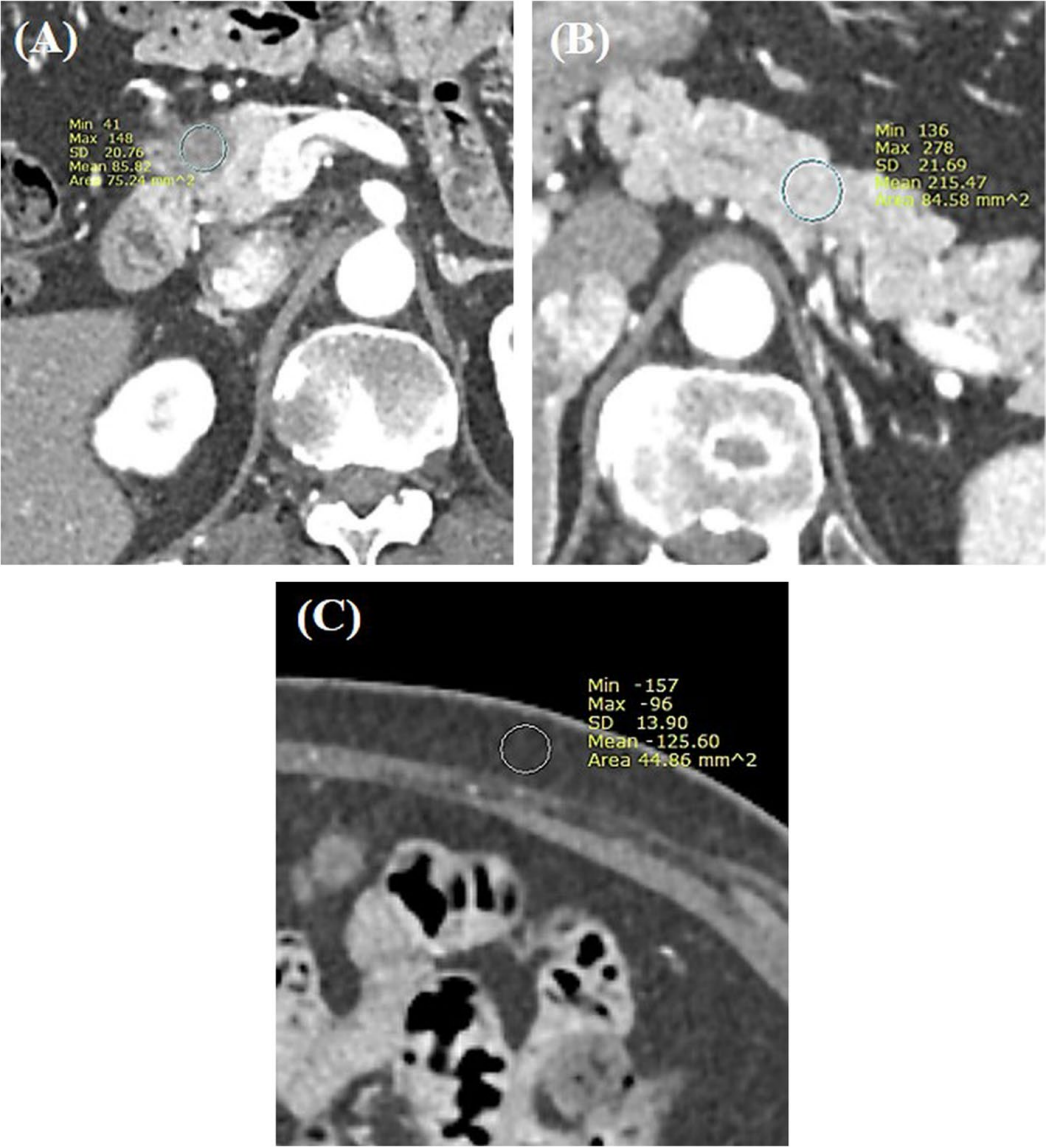
Assessment of CNR for pancreatic ductal adenocarcinoma in a 56-year-old female patient. **A** An arterial phase axial contrast-enhanced CT image from the high-resolution protocol reveals a 2 cm hypo-attenuating lesion with a mean Hounsfield Unit (HU) value of 85.8. **B** The mean HU value for the pancreatic parenchyma was 215.47. **C** Noise level, characterized by the mean standard deviation of subcutaneous fat attenuation, is measured at three distinct locations (one shown here), yielding a value of 13.5. From these measurements, the CNR for the pancreatic ductal adenocarcinoma is calculated to be 9.6

Quantitative measurements were conducted on both the standard protocol and HR-CT scans by one of the authors (D.H.L), who possesses a 10-year of experience in abdominal imaging. The attenuation values of the pancreatic tumor and parenchyma in arterial phase images were meticulously assessed. This involved manually outlining regions of interest (ROIs) on the axial CT images, encompassing as much of the tumor and parenchyma as feasible. Careful attention was paid to avoid areas featuring cystic degeneration, necrosis, adjacent vessels, or dilated ducts during ROI selection. The attenuation values for both the pancreatic tumor and parenchyma were measured three times, with the average of these measurements subsequently utilized for further analysis. The parenchyma-to-tumor contrast-to-noise ratio (CNR) was formulated and computed according to the subsequent equation (Figure 1):

CNR = (Mean pancreas parenchyma – Mean pancreatic tumor) / noise.

The noise was defined as the mean standard deviation value of subcutaneous fat attenuation measured in the anterior abdominal wall [12]. The attenuation of subcutaneous fat was measured three times and then averaged. Alongside measuring the attenuation value of the pancreas tumor and parenchyma for CNR calculation, the mean CT attenuation values of the abdominal aorta and main portal vein (MPV) were also obtained from arterial phase and portal venous axial images. This measurement process was performed for both the standard protocol and HR-CT.

#### Assessment of PDAC resectability

Three board-certified abdominal radiologists evaluated both the standard protocol and HR-CT: S.J.P with 4 years of experience, C.W with 8 years of experience, and M.H.Y with 10 years of experience in abdominal imaging. To ascertain PDAC resectability for each participant, data concerning the tumor's location, size, tumor vascular contact, and distant metastasis were collected and recorded. The resectability of PDAC was determined and categorized into three classes as per recent NCCN guidelines: resectable, borderline resectable, and locally advanced [2], based on tumor vascular contact's presence and extent. Additionally, the likelihood of achieving R0 resection was assessed on both standard protocol and high-resolution pancreas CT using a 5-point scale: 1 for definite likelihood, 2 for probable likelihood, 3 for indeterminate, 4 for probable unlikelihood, and 5 for definite unlikelihood. In accordance with the determined PDAC resectability per NCCN guidelines, a score of 1 was assigned for R0 resection probability to resectable PDAC cases. Similarly, a score of 5 was assigned to locally advanced PDAC cases. Borderline resectable PDAC cases were assigned a score ranging from 2 to 4 based on the degree of tumor vascular contact.

### Determination of PDAC resectability: Reference standard

PDAC resectability was primarily validated through surgical and histopathologic examinations, serving as the reference standard. Drawing from surgical records and histopathologic examinations, we categorized the resection margin status into three groups: R0 indicated no residual tumors on microscopic assessment; R1 implied no gross residual tumor, but the presence of tumor cells within 1 mm of the resection margin or at the cut-surface margin [13]; and R2 denoted the presence of gross residual tumor. Complete tumor removal was classified as R0 resection. If PDAC itself wasn't surgically removed due to occult distant metastasis or local invasion of major vessels, resulting in palliative bypass surgery, it was regarded as unresectable PDAC. Furthermore, participants with locally advanced PDAC on CT who did not undergo surgery after multidisciplinary conference deliberations were classified as having clinically confirmed unresectable PDAC [14].

### Statistical analysis

Continuous variables were presented using the medians along with the interquartile ranges (IQRs). We employed the Mann-Whitney U test or the Wilcoxon signed rank test for comparing continuous variables, and Fisher’s exact test to assess categorical variables. To appraise the diagnostic performance of both the standard protocol and high-resolution pancreas CT in predicting R0 resection for PDAC, we utilized the area under the receiver operating characteristic curve (AUC). Sensitivity, specificity, and accuracy for predicting R0 resection were also computed for both protocols, and their comparison was performed using the McNemar test. For the computation of sensitivity, specificity, and accuracy in predicting R0 resection, we treated scores 1, 2, and 3 of R0 resection probability as indicative of R0 resection. Furthermore, the inter-reader agreement in assessing R0 resection probability was gauged using kappa statistics, categorized as poor (<0.20), fair (0.20–0.40), moderate (0.40–0.60), good (0.60–0.80), and excellent (0.80–1.00) agreements [15]. Statistical analyses were performed using commercially available software programs (MedCalc version 18.9.1, MedCalc Software, Ostend, Belgium).

## Results

### Patients’ characteristics

This study constituted a retrospective subgroup analysis of data derived from a prospective multicenter study that assessed the diagnostic performance of HR pancreas CT in evaluating PDAC resectability (Study identifier: NCT03895177). In the previous multicenter prospective study, 138 individuals with PDAC were initially enrolled and scrutinized. Among them, 69 PDAC patients were excluded from this study due to the absence of available standard protocol pancreas CT scans taken within a month from determining PDAC resectability. Subsequently, the remaining 69 patients (37 males and 32 females), with a median age of 66.5 years (IQR, 61.0-74.0 years), were ultimately included in this study. Within this group, 27 patients (39.1%) underwent neoadjuvant therapy using either chemotherapy combined with radiation therapy (*n*=5) or chemotherapy alone (*n*=22). The baseline characteristics of all patients were summarized in Table 1.

**Table 1:** Patient characteristics

**Characteristics**	
**No. of patients**	69
**Age (years), median (IQR)**	66.5 (61.0-74.0)
**Sex (M:F)**	37:32
**Tumor size (cm), median (IQR)**	2.6 (2.1-3.3)
**Tumor location**	
Head/uncinate	45
Neck/body	15
Tail	9
**Resection margin status**	
R0	51
R1 or R2	18
**Neoadjuvant therapy**	
Yes	42
No	27

*IQR* Interquartile range

### Radiation dose of each CT protocol

The median radiation dose of HR pancreas CT was 11.4 mSv (IQR, 10.3-12.5). The median radiation dose of standard pancreas CT was 14.3 mSv (IQR, 12.0-17.6), which was significantly higher than 11.4 mSv of HR pancreas CT (*P*=0.036).

### Quantitative assessment

**Fig. 2:**
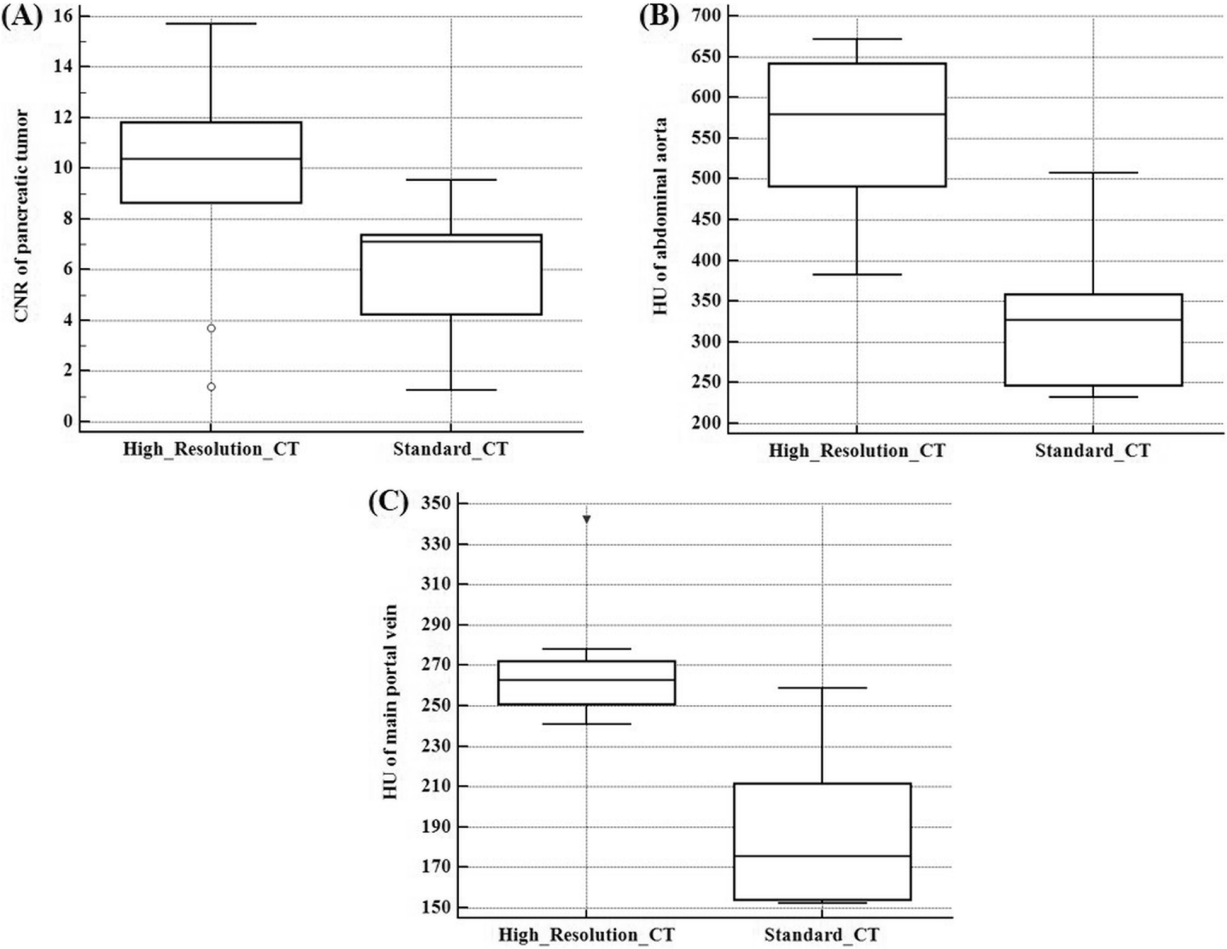
Quantitative analysis results for (**A**) the CNR of pancreatic ductal adenocarcinoma; (**B**) the attenuation value of the abdominal aorta; and (**C**) the attenuation value of the main portal vein. The central box represents the interquartile range while the middle line represents the median value

**Fig. 3:**
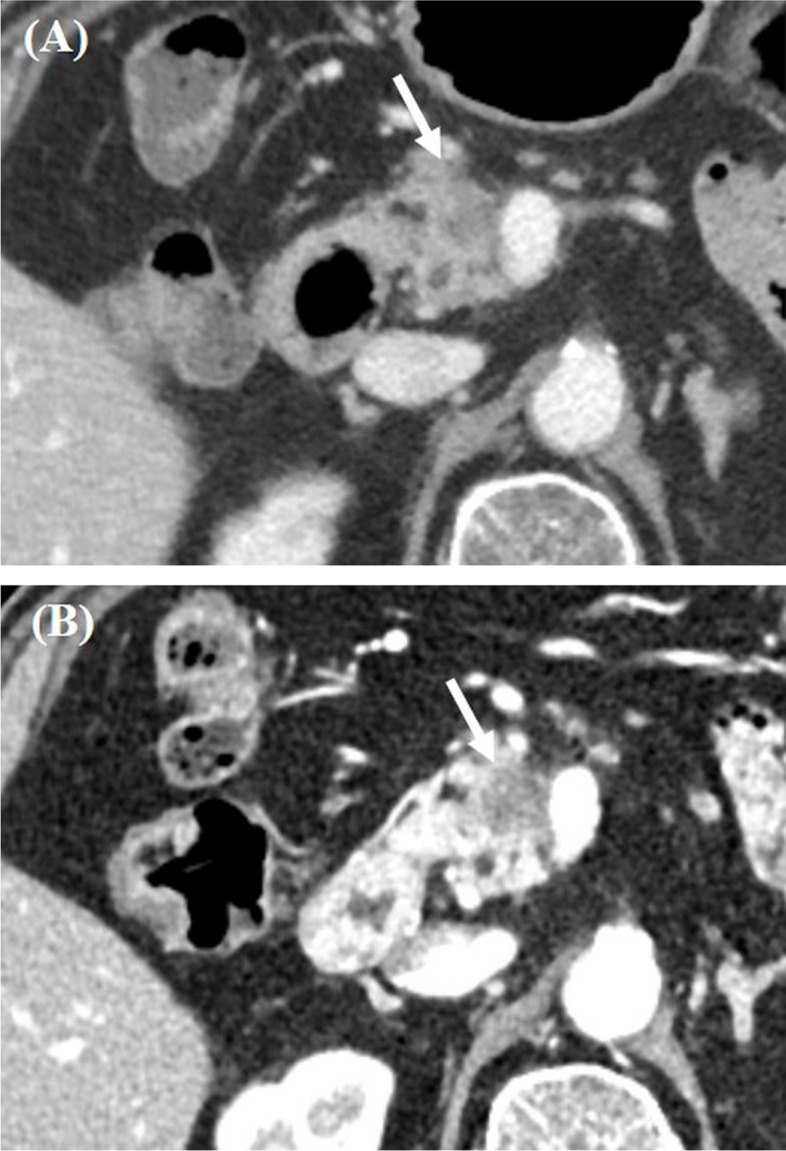
CT images obtained from a 79-year-old man with pancreatic ductal adenocarcinoma in the pancreas head. **A** Arterial phase axial contrast enhanced CT image (window width, 400HU; window level, 40HU) acquired through the standard protocol showed a 1.5 cm hypo-attenuating lesion in the pancreas head (arrow) abutting the SMV. The calculated CNR of pancreatic tumor was 7.4. **B** Arterial phase axial contrast enhanced CT image (window width, 400HU; window level, 40HU) acquired through the high-resolution protocol also revealed a 1.5 cm hypo-attenuating lesion (arrow) with calculated CNR of pancreatic tumor of 15.7. The abutment of SMV was also noted. Upon reviewing these imaging findings, all three readers classified this patient as having resectable pancreatic ductal adenocarcinoma on both CT protocols. Nevertheless, surgical exploration unveiled unexpected peritoneal seeding, making the tumor unresectable

The median CNR of PDAC was 7.1 (IQR, 4.3-7.4) on the standard protocol pancreas CT using 120 kVp, and 10.4 (IQR, 8.7-11.8) on HR-CT using 80 kVp, respectively. This difference was statistically significant (*P*=0.006) (Figure 2 and Figure 3). The median attenuation value of the abdominal aorta measured 579.5 HU (IQR, 491.0-641.0 HU) on HR-CT, which was significantly higher than 327.2 HU (IQR, 247.3-357.9 HU) on the standard protocol pancreas CT (*P*=0.002) (Figure 1). HR-CT also provided significantly higher attenuation values for the MPV (Median, 263.0 HU; IQR, 251.0-272.0 HU) compared to the standard protocol pancreas CT (Median, 175.6 HU; IQR, 154.3-211.7 HU) (*P*=0.004) (Figure 1).

### Assessment of PDAC resectability

Among the 69 patients, five patients did not undergo pancreas resection for the following reasons: the presence of liver metastasis on preoperative CT (*n*=2); the presence of superior mesenteric artery (SMA) invasion on preoperative CT (*n*=1); and the presence of occult distant metastases (*n*=2; liver metastasis in one patient and peritoneal seeding in the other patient) found intraoperatively. Thus, these five patients were considered as having clinically confirmed unresectable PDAC. Among the 64 patients who underwent pancreatic resection with curative intent, R0 resection was achieved in 51 patients (79.7%), and R1 resection was performed in 13 patients (20.3%). In the overall patient cohort, the R0 resection rate was 73.9% (51/69).

The PDAC resectability categories, assessed using both the standard protocol and HR-CT by three readers, along with their corresponding R0 resection rates, are presented in Table 2. In summary, there were no significant differences in the R0 resection rates for each PDAC resectability category between the two pancreas CT protocols across all three readers. The diagnostic performance of each pancreas CT protocol in predicting R0 resection, including the AUC, sensitivity, specificity, and accuracy, is summarized in Table 3. The pooled AUC for HR-CT was 0.727, which was slightly higher than the AUC of 0.699 for the standard protocol pancreas CT. However, this difference was not statistically significant (*P*=0.128). Regarding sensitivity in predicting R0 resection, standard CT exhibited a slightly higher pooled sensitivity compared to HR-CT, although this difference was not statistically significant (79.1% for standard CT vs. 73.2% for high-resolution CT; *P*=0.096). Conversely, HR-CT tended to offer a higher pooled specificity in predicting R0 resection compared to the standard protocol CT, but this difference did not achieve statistical significance (42.6% for standard CT vs. 61.1% for high-resolution CT; *P*=0.081). Furthermore, no significant difference was observed in the pooled accuracy for predicting R0 resection between the two pancreas CT protocols (69.6% for standard CT vs. 70.0% for high-resolution CT; *P*=0.834).

**Table 2:** Margin-negative (R0) resection rate and CT resectability status assessed on each pancreas CT protocol

**CT resectability**		Resection margin status	Standard protocol CT	High-resolution CT	*P*-value
**Reader 1**	**Resectable**	R0	25/28 (89.3%)	22/25 (88.0%)	0.883
		R1 or R2	3/28 (10.7%)	3/25 (12.0%)	0.883
	**Borderline resectable**	R0	14/20 (70.0%)	16/20 (80.0%)	0.465
		R1 or R2	6/20 (30.0%)	4/20 (20.0%)	0.465
	**Locally advanced**	R0	12/21 (57.1%)	13/24 (54.2%)	0.841
		R1 or R2	9/21 (42.9%)	11/24 (45.8%)	0.841
**Reader 2**	**Resectable**	R0	35/40 (87.5%)	29/32 (90.6%)	0.675
		R1 or R2	5/40 (12.5%)	3/32 (9.4%)	0.675
	**Borderline resectable**	R0	9/13 (69.2%)	11/19 (57.9%)	0.515
		R1 or R2	4/13 (30.8%)	8/19 (42.1%)	0.515
	**Locally advanced**	R0	7/16 (43.8%)	11/18 (61.1%)	0.311
		R1 or R2	9/16 (56.2%)	7/18 (38.9%)	0.311
**Reader 3**	**Resectable**	R0	29/33 (87.9%)	27/29 (93.1%)	0.488
		R1 or R2	4/33 (12.1%)	2/29 (6.9%)	0.488
	**Borderline resectable**	R0	12/18 (66.7%)	8/15 (53.3%)	0.435
		R1 or R2	6/18 (33.3%)	7/15 (46.7%)	0.435
	**Locally advanced**	R0	10/18 (55.6%)	16/25 (64.0%)	0.576
		R1 or R2	8/18 (44.4%)	9/25 (36.0%)	0.576

**Table 3:** Diagnostic performance of each pancreas CT protocol in predicting margin-negative (R0) resection

		**Standard CT**	**High-resolution CT**	***P*-value**
**Reader 1**	AUC	0.714 (0.593, 0.816)	0.739 (0.619, 0.837)	0.646
	Sensitivity	76.5% (39/51)	72.5% (37/51)	0.500
	Specificity	50.0% (9/18)	72.2% (13/18)	0.219
	Accuracy	69.6% (48/69)	72.5% (50/69)	0.727
**Reader 2**	AUC	0.702 (0.580, 0.806)	0.728 (0.607, 0.828)	0.682
	Sensitivity	86.3% (44/51)	82.4% (42/51)	0.500
	Specificity	33.3% (6/18)	38.9% (7/18)	0.999
	Accuracy	72.5% (50/69)	71.0% (49/69)	0.999
**Reader 3**	AUC	0.680 (0.557, 0.787)	0.714 (0.593, 0.816)	0.359
	Sensitivity	74.5% (38/51)	64.7% (33/51)	0.125
	Specificity	44.4% (8/18)	72.2% (13/18)	0.063
	Accuracy	66.7% (46/69)	66.7% (46/69)	0.999
**Pooled analysis**	AUC	0.699 (0.582, 0.816)	0.727 (0.605, 0.849)	0.128
	Sensitivity	79.1% (66.8%, 91.3%)	73.2% (56.7%, 89.7%)	0.096
	Specificity	42.6% (21.0%, 64.2%)	61.1% (24.9%, 97.4%)	0.081
	Accuracy	69.6% (60.3%, 78.8%)	70.0% (61.4%, 83.4%)	0.834

*AUC* Area under the receiver operating characteristic curve

**Fig. 4:**
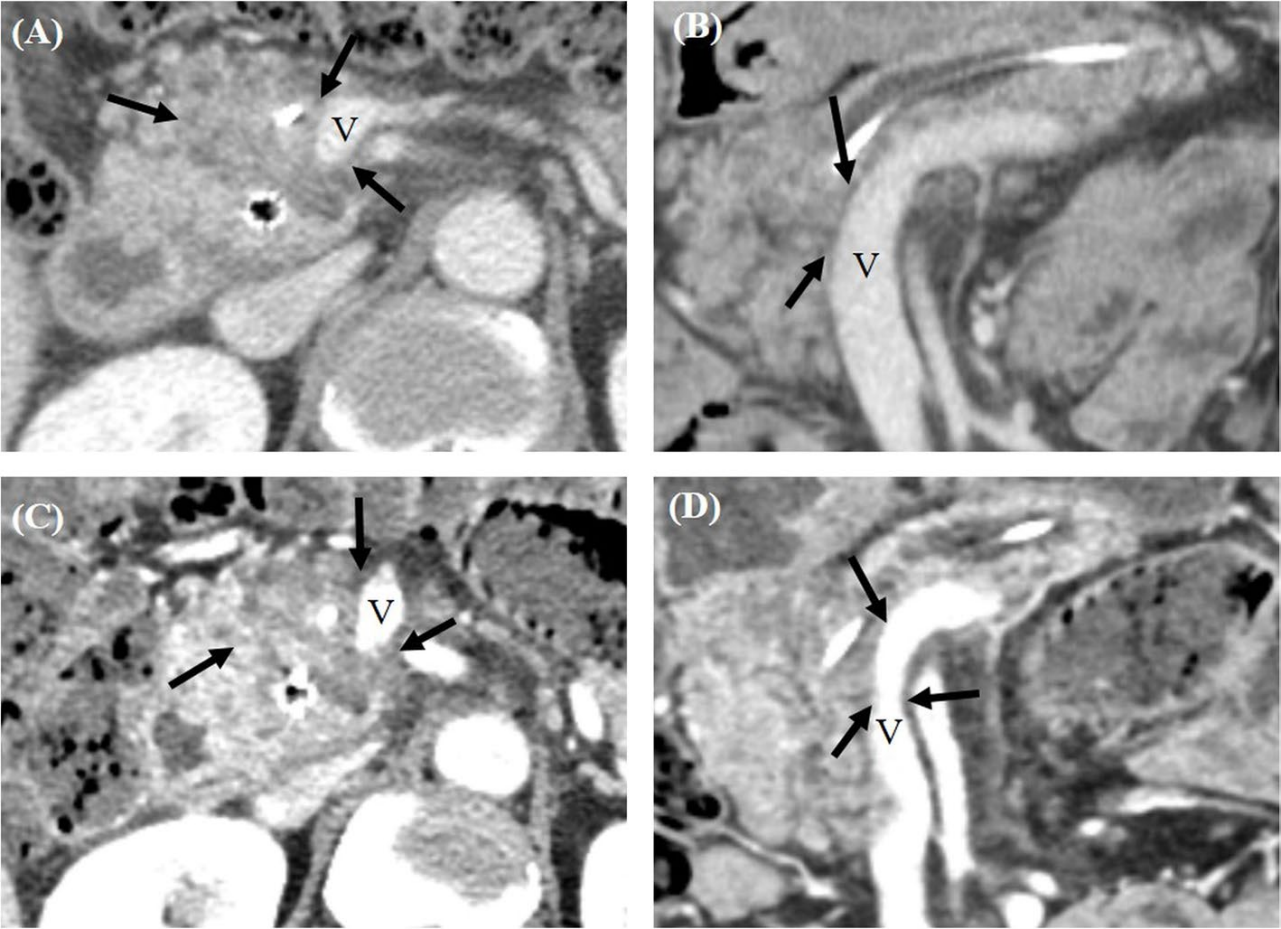
CT images from a 57-year-old male with pancreatic ductal adenocarcinoma in the pancreas head. **A** Portal venous phase axial contrast-enhanced CT image (window width, 400 HU; window level, 40 HU) obtained using the standard protocol displaying a 3.5 cm hypo-attenuating lesion in the pancreas head (arrows) abutting both the SMV and SMA. **B** Portal venous phase coronal CT image obtained through the standard protocol also revealed abutment of the pancreatic ductal adenocarcinoma to the SMV. Based on these imaging findings, all three reviewers initially categorized this patient as having borderline resectable pancreatic ductal adenocarcinoma. **C** Using the high-resolution protocol, a portal venous phase axial contrast-enhanced CT image (window width, 400 HU; window level, 40 HU) also identifies the 3.5 cm hypo-attenuating lesion (highlighted by an arrow) abutting the SMV and SMA. **D** Notably, the high-resolution coronal CT image reveals luminal narrowing of the SMV due to tumor encasement (indicated by arrows) with an involved length of 2.7 cm. Based on this, two out of the three reviewers reclassified the patient's condition as locally advanced pancreatic ductal adenocarcinoma. While a surgical resection was undertaken, the post-operative histopathologic examination confirmed an R1 resection

The diagnostic performance of each pancreas CT protocol in predicting R0 resection based on the history of neoadjuvant therapy is summarized in Table 4. Both CT protocols demonstrated a higher pooled AUC for patients undergoing upfront surgery compared to those who received neoadjuvant therapy (Standard CT: 0.766 vs. 0.541; HR-CT: 0.827 vs. 0.533). Among the 42 patients who underwent upfront surgery, HR-CT significantly outperformed standard CT in terms of specificity for R0 resection (70.4% vs. 44.4%, *P*=0.016). However, other metrics, including AUC, sensitivity, and accuracy in predicting R0 resection, showed no significant differences between the two CT protocols. The representative cases were given in Figure 3 and Figure 4.

**Table 4:** Diagnostic performance of each pancreas CT protocol in predicting margin-negative (R0) resection according to the history of neoadjuvant therapy

	**Upfront surgery (*n*=42)**				**Neoadjuvant therapy (*n*=27)**			
		**Standard CT**	**High-resolution CT**	***P*-value**		**Standard CT**	**High-resolution CT**	***P*-value**
**Reader 1**	AUC	0.739 (0.581, 0.862)	0.842 (0.695, 0.936)	0.157	AUC	0.520 (0.415, 0.799)	0.580 (0.376, 0.766)	0.569
	Sensitivity	87.9% (29/33)	84.8% (28/33)	0.999	Sensitivity	55.6% (10/18)	50.0% (9/18)	0.999
	Specificity	44.4% (4/9)	77.8% (7/9)	0.250	Specificity	55.6% (5/9)	66.7% (6/9)	0.999
	Accuracy	78.6% (33/42)	83.8% (35/42)	0.625	Accuracy	55.6% (15/27)	55.6% (15/27)	0.999
**Reader 2**	AUC	0.744 (0.586, 0.866)	0.865 (0.724, 0.951)	0.162	AUC	0.596 (0.391, 0.779)	0.512 (0.314, 0.708)	0.383
	Sensitivity	93.9% (31/33)	93.9% (31/33)	0.999	Sensitivity	72.2% (13/18)	61.1% (11/18)	0.500
	Specificity	33.3% (3/9)	55.6% (5/9)	0.500	Specificity	33.3% (3/9)	44.4% (4/9)	0.999
	Accuracy	81.0% (34/42)	85.7% (36/42)	0.500	Accuracy	59.3% (16/27)	55.6% (15/27)	0.999
**Reader 3**	AUC	0.835 (0.688, 0.931)	0.832 (0.684, 0.929)	0.894	AUC	0.552 (0.350, 0.742)	0.512 (0.314, 0.708)	0.560
	Sensitivity	84.8% (28/33)	81.8% (27/33)	0.999	Sensitivity	55.6% (10/18)	33.3% (6/18)	0.125
	Specificity	55.6% (5/9)	77.8% (7/9)	0.500	Specificity	33.3% (3/9)	66.7% (6/9)	0.250
	Accuracy	78.6% (33/42)	81.0% (34/42)	0.999	Accuracy	48.1% (13/27)	44.4% (12/27)	0.999
**Pooled analysis**	AUC	0.766 (0.682, 0.837)	0.827 (0.749, 0.888)	0.119	AUC	0.541 (0.427, 0.652)	0.533 (0.419, 0.645)	0.856
	Sensitivity	88.9% (82.6%, 95.2%)	86.9% (80.1%, 93.6%)	0.625	Sensitivity	59.3% (45.7%, 72.8%)	48.2% (34.4%, 61.9%)	0.061
	Specificity	44.4% (24.4%, 64.5%)	70.4% (52.0%, 88.8%)	0.016	Specificity	44.4% (24.4%, 64.5%)	59.3% (39.5%, 79.1%)	0.219
	Accuracy	79.4% (72.2%, 86.5%)	83.3% (76.7%, 89.9%)	0.227	Accuracy	54.3% (43.2%, 65.4%)	51.9% (40.7%, 63.0%)	0.774

*AUC* Area under the receiver operating characteristic curve

### Inter-reader agreement

Inter-reader agreements on the assessment of R0 resection probability, as evaluated using both pancreas CT protocols, are presented in Table 5. The kappa values ranged from 0.588 to 0.653 for the standard pancreas CT protocol, indicating moderate to good inter-reader agreement. Similarly, the HR-CT also demonstrated moderate to good agreement, with kappa values ranging from 0.596 to 0.722.

**Table 5:** Inter-reader agreement of each pancreas CT protocol in assessing the probability of margin-negative (R0) resection assessed by κ value

Inter-reader agreement	Readers 1 and 2	Readers 2 and 3	Readers 3 and 1
Standard protocol CT	0.588 (0.450-0.726)	0.653 (0.489-0.816)	0.642 (0.492-0.791)
High-resolution CT	0.596 (0.462-0.730)	0.722 (0.620-0.825)	0.659 (0.523-0.795)

## Discussion

For patients with PDAC, surgery complemented by perioperative chemotherapy offers the best chance for long-term survival. Considering the significant morbidity associated with PDAC surgery, accurate staging becomes essential [16, 17]. In this study, HR-CT employing an 80 kVp tube voltage and a 1 mm ST significantly outperformed the standard CT protocol, which utilized a 120 kVp tube voltage and 3 mm ST, achieving a higher CNR for PDAC (10.4 for HR-CT vs. 7.1 for standard CT, *P*=0.006). Additionally, when comparing median attenuation values, the abdominal aorta and MPV showed better results on HR-CT using 80 kVp than on the standard CT. Nevertheless, a surprising observation was that despite the heightened CNR for PDAC with HR-CT, the anticipated enhancement in evaluating tumor vascular contact—a critical aspect in determining PDAC resectability—did not manifest. This finding was further emphasized by the statistically insignificant difference in the pooled AUC for predicting R0 resection between HR-CT and the standard CT (*P*=0.128). These findings underscore the need for further investigation into the factors influencing the diagnostic performance of HR-CT in the context of PDAC resectability, especially given its technical superiority in image quality.

In our study, with the aim of enhancing both contrast and spatial resolution for pancreas protocol CT, we employed an HR-CT protocol set at 80 kVp and a 1 mm ST with a 1 mm interval, along with model-based iterative reconstruction. Since the mean effective energy of the X-ray spectrum from an 80 kVp X-ray tube aligns more closely with the iodine K-edge compared to a 120 kVp voltage, HR-CT at 80 kVp can achieve more pronounced contrast enhancement with iodinated material than standard CT at 120 kVp [10]. This principle helps explain our findings, where the contrast enhancement in the abdominal aorta and MPV using HR-CT surpassed that of the standard CT. Additionally, an increase in contrast enhancement is directly linked to improved PDAC CNR. Past research indicates that low tube voltage CT at 80 kVp can achieve approximately a 10% enhancement in CNR compared to standard CT at 120 kVp [10, 12]. Our study's findings were consistent with those of previous studies.

In our study, HR-CT using 80 kVp showed a superior CNR for PDAC compared to the standard CT at 120 kVp. This theoretically should facilitate enhanced PDAC tumor delineation and improved evaluation of tumor vascular contact — a pivotal factor in determining PDAC resectability. However, the observed outcomes fell short of our expectations. Specifically, while the pooled AUC of HR-CT for predicting R0 resection of PDAC stood at 0.727, slightly exceeding the 0.699 of standard CT, the difference was not statistically significant (*P*=0.128). Moreover, both CT protocols displayed comparable sensitivity, specificity, and accuracy in their predictions. Earlier research noted that a 1 mm reconstruction interval on thin-section CT boosted the detectability of perineural invasion by both PDAC and extrahepatic bile duct cancer [11, 18]. One plausible explanation for the unexpected R0 prediction outcomes in our HR-CT study could be the difficulty in differentiating true tumor infiltration from desmoplastic reactions and the accompanying fibrosis or inflammation. These presentations frequently exhibit an indistinguishable appearance on CT images. Moreover, this challenge is compounded by post-neoadjuvant therapy for PDAC, which introduces additional variations such as tumor regression and inflammatory changes, thereby amplifying the complexity of discernment, even in light of the resolution advantages provided by HR-CT [2, 19–21]. Supporting this, our data demonstrated a diminished pooled AUC for HR-CT in predicting R0 resection among patients who underwent post-neoadjuvant therapy, in alignment with earlier studies. This highlights potential uncertainties surrounding the precision of CT-identified tumor vascular contacts after neoadjuvant interventions. Thus, elevating the image quality of pancreas protocol CT in isolation might prove insufficient. Complementary criteria, reflecting tumor biology or metabolism, could be imperative for accurately assessing PDAC resectability, especially post-neoadjuvant therapy [14].

Our study is subject to several limitations. First, as this study was a retrospective analysis of a prospectively constructed cohort from a previous multicenter study, the possibility of selection bias cannot be disregarded. Second, the relatively small number of PDAC patients in our study (*n*=69) restricted the statistical power. While our study indicated that HR-CT tended to perform better in predicting R0 resection compared to the standard protocol CT, the statistically significant difference was not found, likely due to the small number of patients. Third, our study exclusively included relatively lean patients with a body mass index (BMI) ranging from 17.5 kg/m^2^ to 28.1 kg/m^2^. It is well established that the utilization of low tube voltage in obese patients, especially those with a BMI of ≥30 of kg/m^2^, can lead to heightened image noise, potentially compromising image quality. Consequently, future studies adopting a prospective design and encompassing a larger patient cohort, including those with higher BMI, are imperative to corroborate our study's findings.

## Conclusion

The application of HR-CT using 80 kVp demonstrated a notably elevated level of contrast enhancement in vessels and CNR for PDAC, surpassing that of the standard CT. Nevertheless, despite these enhancements, HR-CT did not yield an improvement in diagnostic performance for predicting R0 resection when compared to standard CT.

## Data Availability

The data will be available per reasonable request to the corresponding author after approval of IRB for data sharing.

## Abbreviations

PDAC: Pancreatic ductal adenocarcinoma
CT: Computed tomography
NCCN: National Comprehensive Cancer Network
HR: High resolution
ROI: Regions of interest
CNR: Contrast-to-noise ratio
MPV: Main portal vein
IQR: Interquartile range
AUC: Area under the receiver operating characteristic curve
